# Effect of Yttrium on the Microstructure and Mechanical Properties of PH13-8Mo Stainless Steels Produced by Selective Laser Melting

**DOI:** 10.3390/ma15155441

**Published:** 2022-08-08

**Authors:** Chang-Jun Wang, Chang Liu, Meng-Xing Zhang, Lu Jiang, Yu Liu, Zhen-Bao Liu, Jian-Xiong Liang

**Affiliations:** 1 Central Iron & Steel Research Institute Company Limited, Beijing 100081, China; 2 Institute for Frontier Materials, Deakin University, Geelong, VIC 3216, Australia

**Keywords:** high-strength stainless steel, selective laser melting, rare earth modification, yttrium

## Abstract

In the present work, PH13-8Mo stainless steel parts without yttrium and with yttrium (Y) were manufactured by selective laser melting (SLM). The microstructure, phase composition and grain orientation of the stainless steels parts with Y and without Y were characterized by scanning electron microscopy (SEM), X-ray diffraction (XRD), electron-backscatter diffraction (EBSD), transmission electron microscopy (TEM) and high-resolution transmission electron microscopy (HRTEM). The characterization results revealed that the addition of Y clearly refined the grain size of the PH13-8Mo steel formed part, resulting in more equiaxed massive grains and in a less anisotropic microstructure. PH13-8Mo stainless steel formed parts were mainly composed of martensite and retained austenite. The addition of Y could significantly increase the content of retained austenite and also generate nano-sized precipitates containing Y. The mechanical test results showed that both strength and toughness of the shaped parts containing Y were improved synergistically. The yield strength reached 1443 MPa, the elongation was 12.2%, and the room temperature impact energy reached 124.25 J/cm^2^. The strengthening and toughening by Y of the formed parts were mainly attributed to grain refinement, higher volume fraction of the retained austenite and the formation of nano-sized precipitates containing Y.

## 1. Introduction

PH13-8Mo stainless steel is a martensitic precipitation hardening stainless steel with excellent properties. Because of its high strength and toughness, good corrosion resistance, formability and weldability, it has been widely used in the marine, aerospace, nuclear reactor, petrochemical industries and other fields [1,2,3]. However, with the expansion of application fields and the improvement of the complexity of stainless steel parts, the traditional smelting and forging processes cannot meet the current needs. Fortunately, additive manufacturing technology, which has attracted extensive attention in recent years, has effectively solved the problem of the difficult processing of complex stainless steel parts. Among many additive manufacturing technologies, selective laser melting (SLM) is the most common 3D printing technology for metal materials. Selective laser melting technology uses a high-energy laser beam to selectively melt a metal powder layer by layer and manufactures three-dimensional parts by spreading the powder layer by layer and then melting, solidifying and stacking it layer by layer. It has been widely used for stainless steel, titanium alloys, high-entropy alloys, nickel-based alloys and other materials [4,5,6].

In recent years, great progress has been made in research on additive manufacturing of PH13-8Mo stainless steels. Dong et al. [7] adjusted the SLM process laser energy density(η) to 245 J/m and obtained dense PH13-8Mo stainless steel SLM forming parts, with the optimum density reaching 99.72%, which provided process guidance for the formation by SLM of PH13-8Mo stainless steel parts. The mechanical properties of PH13-8Mo stainless steels formed by SLM were tested. It was found that the ultimate tensile strength reached 1068 MPa, and the elongation after fracture reached 15.7%. Although the stainless steel parts formed by SLM showed superior plastic toughness, the strength of PH13-8Mo stainless steel SLM parts not subjected to heating cannot meet the mechanical standard requirements of high-strength stainless steels (>1400 MPa). This problem also exists in different additive manufacturing processes. Hamed Asgari et al. [8] used direct metal laser sintering (DMLS) to obtain a nearly fully dense PH13-8Mo stainless steel with a microstructure of fine lath martensite. The mechanical test results showed that the elongation at break of DMLS-formed parts without heat treatment reached 21.7%, showing excellent plastic toughness. However, their tensile strength was only 1113 MPa. In order to improve the tensile strength of the additive manufactured PH13-8Mo stainless steels, Yan et al. [9] produced a dense PH13-8Mo stainless steel by the SLM process, with a microstructure mixed with fine martensite, retained austenite and rod-shaped NiAl nano-sized precipitates. This was similar to the microstructure of the PH13-8Mo stainless steel obtained by traditional melting methods, but the grain size was obviously refined due to the rapid cooling characteristics of the SLM process. In Yan’s work, the strength of the PH13-8Mo stainless steel formed by SLM reached 1528 MPa after heat treatment. However, the plastic toughness was significantly deteriorated, and the elongation after fracture was reduced to 7.3%. Therefore, the problem of strength and plastic constraints of PH13-8Mo stainless steel SLM-formed parts was not solved.

Toughness reduction is a common issue in SLM-formed stainless steels. The main reason is that SLM is a cooling forming process involving the melting of stainless steel powder through laser scanning. The oxygen content in the powder raw material is significantly higher than that in the molten steel, resulting in a large amount of oxygen impurities in the subsequent SLM-formed parts. Oxygen atoms impurities will combine with alloying elements in stainless steels to form brittle oxide inclusions, such as Al_2_O_3_. This is the most important factor reducing the plastic toughness of stainless steel formed parts [10,11]. At present, the solution is to improve the quality of raw material powder, that is, to use high-quality powder with extremely low oxygen content as SLM raw material. However, the price of low-oxygen powder is very high, which greatly increases the production cost and does not allow the wide application of SLM to obtain stainless steels. Therefore, at this stage, it is urgent to find a low-cost and effective method to improve the plasticity and toughness of stainless steel made by SLM.

In this work, we newly developed a facile pathway to obtain rare earth-modified stainless steel with improved plastic toughness. Rare earth modification is a common way to improve the properties of stainless steel in the traditional stainless steel smelting process. For example, R. B. Tuttle et al. [12] found that brittle inclusions in stainless steel were reduced, and the strength and plastic toughness of the material were improved by adding the rare earth Ce to 4130 steel. However, there are few reports on how to improve the properties of SLM stainless steel parts by adding rare earth elements, and there is also a lack of in-depth studies on the mechanism of rare earth modification in SLM technology. Our research shows that the addition of rare earth can effectively improve the properties of PH13-8Mo stainless steel SLM-formed parts, leading to high tensile strength and high plastic toughness at the same time. Furthermore, we also analyzed the microstructure, phase composition and precipitation phase changes of PH13-8Mo stainless steel SLM-formed parts after adding a rare earth.

## 2. Materials and Methods

### 2.1. Preparation of Stainless Steel Powder

The powder used in this experiment was prepared by the vacuum induction melting gas atomization method (VIGA). The atomization process was as follows: the discharge temperature was 1740 °C, the gas pressure was 7.2 MPa, and the nozzle diameter was 6 mm. Using a high-purity PH13-8Mo alloy prepared by double vacuum smelting and rare earth yttrium as raw materials, two kinds of PH13-8Mo stainless steel powders without yttrium and with yttrium were prepared, and their compositions are shown in Table 1. We used the elemental composition measured by the ICP-MS method; the device model was plasma 300.

**Figure 1:**
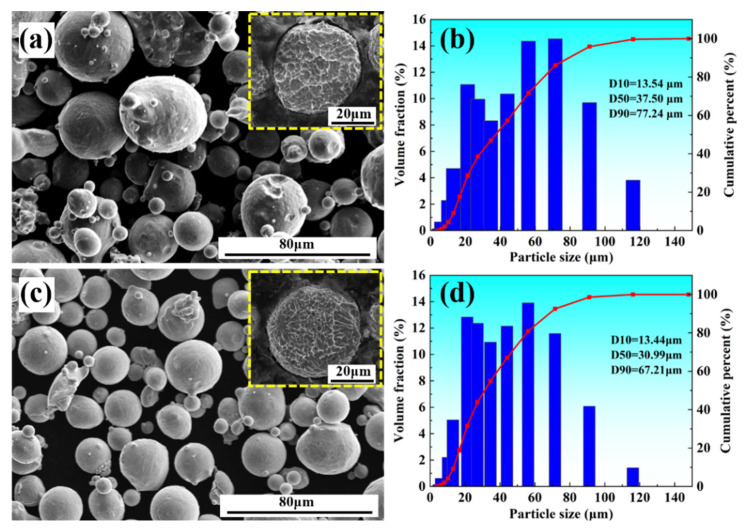
(**a**,**c**) Morphology of the stainless steel powder without and with Y, respectively; (**b**,**d**) particle size distribution of the stainless steel powder without and with yttrium, respectively.

Figure 1 shows the morphology and particle size distribution of powders with different compositions. By observing Figure 1a,c, it can be found that the powder containing Y had a smoother surface, and the surface satellite powder was significantly reduced. From the etching cross section of a single powder particle, it was clearly found that the cross section of the powder presented a cellular structure, and the cellular structure of the powder particles containing yttrium was finer. The particle size distribution of the powder was determined by a laser diffraction particle analyzer (Mastersizer 2000, Malvern instruments Ltd., Malvern, UK). The particle size distribution of the two powders was compared in Figure 1b,d. It was found that the powder without Y had a large particle size, D10 = 13.54 μm, D50 = 37.50 μm, D90 = 77.24 μm. The particle size of powder containing yttrium was smaller, and the particle size distribution curve moved to the left, D10 = 13.44 μm, D50 = 30.99 μm, D90 = 67.21 μm.

### 2.2. SLM Preparation Process and Heat Treatment Process

**Figure 2:**
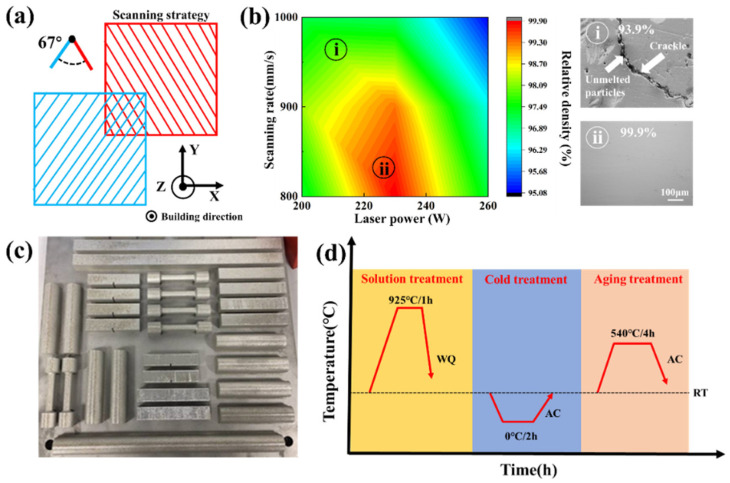
(**a**) Scanning strategy of the SLM forming process; (**b**) comparison of the density of the printed matter for different SLM forming parameters; (**c**) physical drawing of the SLM-formed parts; (**d**) heat treatment of the SLM-formed parts.

The equipment used for SLM forming was PERA China-DLM-280 equipped with a 400W fiber laser with spot size of 100 μm. The wavelength was 1.06 μm. The printing chamber was filled with high-purity argon (Ar 99.99%) as a protective atmosphere to avoid oxidation. According to the published literature on the SLM forming process of stainless steel, the laser scanning strategy for SLM forming adopts a single-pass interlaminar rotation of 67°, as shown in Figure 2a. Before printing the test piece, in order to determine the best printing process window, we set multiple groups of SLM forming parameters for comparison. The parameters are shown in Table 2.

The density of printed matter under different printing parameters is measured by the Archimedes method [13]. The result is presented as an image in Figure 2b. It can be seen in the figure that there were obvious cracks and unmelted powder in the low-density (i) area, while the high-density (ii) area showed a high forming quality and no obvious defects on the surface. Finally, the SLM forming parameters were determined at a laser power of 230 W and a scanning rate of 800 mm/s. The powder prepared by the VIGA method was printed into standard mechanical parts according to the established process parameters, as shown in Figure 2c. After printing, we heat-treated the formed parts according to the heat treatment process shown in Figure 2d. After obtaining a solid solution at 925 °C for 1 h, we cooled the parts to room temperature by water, then cooled them at 0 °C for 4 h, and finally cooled them to room temperature after aging at 540 °C for 4 h.

### 2.3. Characterization of Microstructure and Mechanical Properties

The phase constituents were determined by X-ray diffraction (XRD, Siemens D500, München, Germany) at room temperature and 40 kV. The microstructure and morphology of the samples were observed by scanning electron microscopy (SEM, Zeiss LEO-1450, Oberkochen, Germany) and transmission electron microscopy (TEM, FEI G2, Lincoln, NE, USA) combined with electron diffraction (SAED) analysis of the selected area. An FEI Titan Themis 300 high-resolution TEM (HRTEM), equipped with a probe aberration corrector and a highly efficient energy-dispersive X-ray (EDX) system, was further used for atomic and nanoscale characterization in the scanning mode operating at 300 kV. The texture orientation of the parts was analyzed by electron backscattered diffraction (EBSD, Oxford NordlysNano, Abingdon, UK). The scanning step in the detection of EBSD was 0.03 μm. The samples used for SEM observation were polished employing SiC grinding papers according to a standard metallographic procedure, with the final polishing step using a 0.06 μm colloidal silica solution. Then, each sample was etched for 30 s in Kalling’s solution with 100 mL C_2_H_5_OH, 100 mL HCl and 5 g CuCl_2_ for SEM. The preparation of the EBSD samples involved the use of a KClO_4_ + C_2_H_5_OH (1:9) solution to electropolish the polished small pieces. The polishing voltage was 15 V, the current was 0.6 A, and the time was 15 s. The samples used for TEM and HRTEM observation were fabricated by firstly grinding the sample slices to a thickness of 20–50 μm, followed by milling to 60 nm using a Focused Ion Beam (FIB) device. The phase change points M_s_ and M_f_ of the formed parts were detected by a fully automatic phase change instrument, using the equipment model FORMASTOR-FII

The stainless steel sheets obtained by SLM after heat treatment were cut into dog-bone shaped tensile bars (with a gauge length of 15.0 mm, a center width of 2.0 mm and a thickness of 1.5 mm) and U-port impact samples (with a length of 55.0 mm, a width of 10.0 mm and a thickness of 10.0 mm) using an electric arc wire cutter. The tensile properties of the stainless steels bars were measured on an Instron 5982 machine using an extensometer with a cross-head speed of 1 mm/min at room temperature. Five samples were used to ensure repeatability in each experiment, and the average mechanical properties were determined. The fracture morphology of the tensile samples was evaluated using SEM. The room temperature oscillographic impact was determined using the Ti Nisolsen oscillographic impact testing machine. We prepared two parallel samples for each experiment, too.

## 3. Results

### 3.1. Microstructure Characteristics

**Figure 3:**
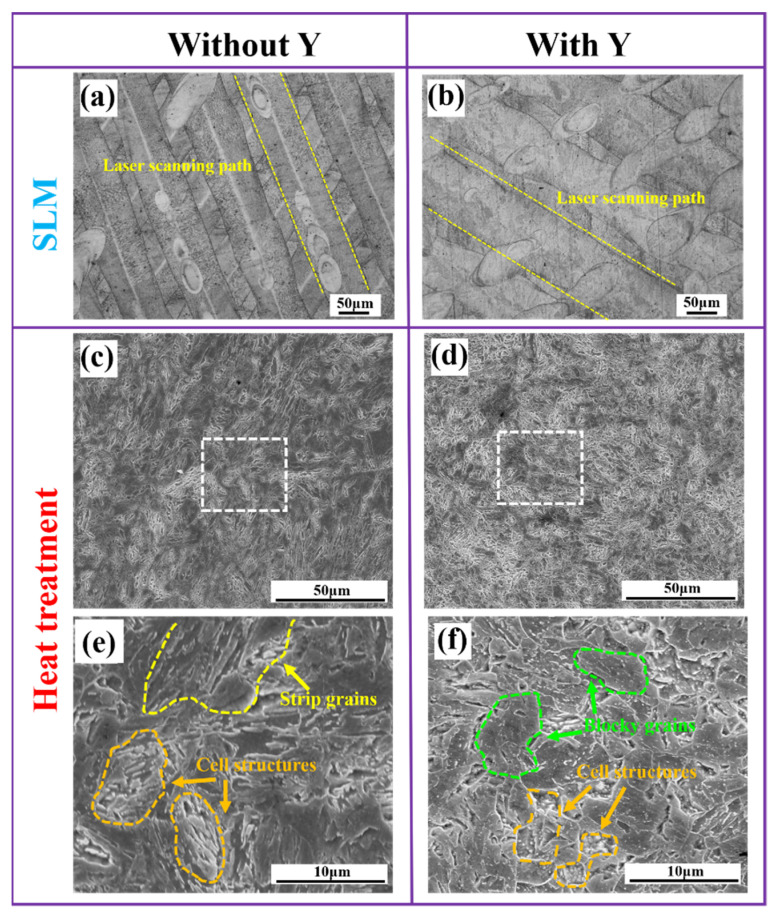
(**a**,**b**) Microstructure of the as-printed stainless steel parts without and with yttrium, respectively; (**c**,**d**) microstructure of the stainless steel formed parts without and with yttrium after heat treatment, respectively; (**e**,**f**) local enlarged areas of (**c**,**d**), indicated by the white dash rectangle.

Figure 3 shows the microstructure of prints with Y and without Y, in two different states, i.e., as-printed and heat-treated. In the as-printed parts, as shown in Figure 3a,b, the laser scanning track could be clearly seen. Because the scanning strategy of interlaminar rotation was adopted in SLM forming in this experiment, the scanning track of the surface was at a certain angle. By comparison, it was found that the surface weld path of the print containing yttrium was wider, with a ladle-like weld pool area. On the contrary, in the as-printed part without yttrium, the weld path was narrow. This could be due to changes in the melting point of the powder. A large number of studies [14,15,16] have shown that the addition of rare earth elements can reduce the melting point of steels. The lower melting point made the powder containing yttrium easier to melt during the laser scanning process, leading to a higher formation efficiency of the molten pool and a longer retention time of the molten pool. This allowed the molten steel to diffuse to a greater extent, so the molten pool area increased.

Figure 3c–f shows the microstructure of the formed part after the heat treatment. Figure 3e,f shows an enlargement of local areas in Figure 3c,d, respectively. It was found that the laser scanning path on the surface of the printed part after the heat treatment disappeared, and the structure of the formed part could be clearly observed. The microstructure of the parts with yttrium addition was obviously finer and more uniform than that of the parts without yttrium. It can be seen from the locally enlarged area that there were cell structures and strip grains in the formed parts without yttrium addition. The cell structure also existed in the formed parts with yttrium, but it was obviously refined compared with that in the formed parts without yttrium. No obvious strip grains were observed in the formed parts with yttrium; instead, massive equiaxed grains were observed.

**Figure 4:**
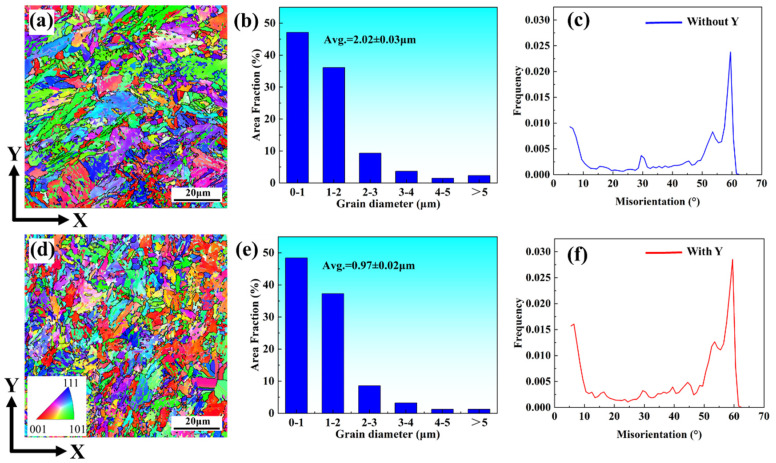
(**a**,**d**) IPF drawing of the formed parts without and with yttrium, respectively; (**b**,**e**) grain size distribution of the formed parts without and with yttrium, respectively; (**c**,**f**) grain boundary angular distribution of the formed parts without and with yttrium.

In order to further observe the effect of yttrium on the grain structure of stainless steel printed parts, EBSD was carried out on the formed parts with Y and without Y after heat treatment, as shown in Figure 4. Through the inversed pole figure (IPF) IPF-z diagram in the X–Y direction (perpendicular to the forming direction), as shown in Figure 4a,d, it was revealed that the grains in the formed parts without yttrium had a certain growth orientation, mainly green, i.e., the <101> direction, indicating that the formed parts had a certain anisotropy. However, the formed parts with yttrium had no specific growth orientation, the grain orientation was dispersed, and the anisotropy was weak. Comparing the grain size distribution (Figure 4b,e), it was found the average grain size of parts without yttrium was 2.02 ± 0.03 mm. The average grain size of parts with yttrium decreased to 0.97 ± 0.02 mm. The EBSD test results further proved that the addition of the rare earth yttrium refined the grain size of the PH13-8Mo stainless steel print. In many studies on rare earth-modified stainless steel, it has been proposed that rare earth elements can refine the grain size, which is also a reason why the addition of rare earth can improve the strength of stainless steel [17,18,19]. By comparing the angular distribution of grain boundaries (Figure 4c,f), it was found that there were more low-angle grain boundaries (5°~10°) in the formed parts with yttrium. This also showed that the addition of yttrium played a role in refining the grains of the formed parts.

### 3.2. Phase Fraction

**Figure 5:**
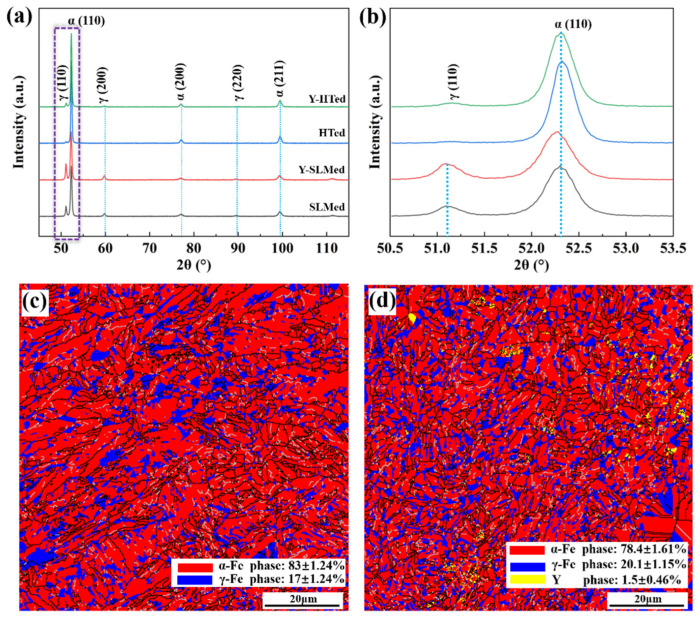
(**a**) XRD patterns of SLM-formed parts with Y and without Y; (**b**) local XRD pattern in (**a**); (**c**) EBSD phase distribution diagram of yttrium-free formed parts; (**d**) EBSD phase distribution diagram of yttrium-containing formed parts.

Figure 5 shows the XRD spectra of the formed parts with Y and without Y in different conditions. It is shown that the main phases of the formed parts were martensite and a small amount of retained austenite. The extremely high cooling rate during SLM limited the diffusion of elements in stainless steel, resulting in a low austenite content. Therefore, martensite was the main phase of PH13-8Mo stainless steels obtained via SLM. It was also found that the diffraction peak of the as-printed stainless steel shifted to a lower angle by comparing the diffraction peak of the as-printed steel with that of heat-treated stainless steel. The literature shows that surface residual stress is the main reason for the left shift of the XRD diffraction peaks [20]. Comparing the diffraction peaks of the formed parts with different yttrium content, we observed that there was no obvious difference in the phase fraction between the two formed parts. In the heat-treated state, the austenite diffraction peak intensity of the formed parts containing yttrium was slightly higher than that of the formed parts without yttrium, and the austenite diffraction peak shifted slightly to the left.

In order to further study the effect of yttrium addition on the phase of stainless steel formed parts, the phases of two formed parts with Y and without Y after heat treatment were scanned by EBSD, as shown in Figure 5c,d. It can be seen that the two formed parts contained a high-martensite phase (red) and a small amount of retained austenite phase (blue), consistent with the XRD results. However, a small amount of yttrium-containing phase (yellow) was also observed in the formed parts containing yttrium, which was mainly distributed along the boundary of martensite and austenite. In terms of phase fraction, the martensite content in the formed parts without yttrium was 83 ± 1.24%, and the austenite content was 17 ± 1.24%. The content of martensite, austenite and yttrium precipitates in the formed parts containing yttrium was 78.4 ± 1.61%, 20.1 ± 1.15% and 1.5 ± 0.46%, respectively. It was found that the addition of yttrium was beneficial as it improved the content of residual austenite in the formed parts. On the one hand, yttrium probably changed the phase transition point of stainless steel, thus affecting the phase transition of the system, which has been reported in many studies on rare earth elements [21,22]. In this work, the addition of yttrium reduced the M_s_ point and M_f_ point of the PH13-8Mo stainless steel (the actual result of phase transformation instrument detection was that they were reduced by 60 °C), thus expanding the austenite phase zone, stabilizing the austenite phase and increasing the austenite content. Another reason may be that martensitic transformation is a displacement-type transformation and is affected by interface energy. The enrichment of rare earth yttrium at the grain boundary reduces the interfacial energy, which weakens the thermal activation condition of martensitic transformation, hinders martensitic transformation, and increases the content of austenite [23,24].

**Figure 6:**
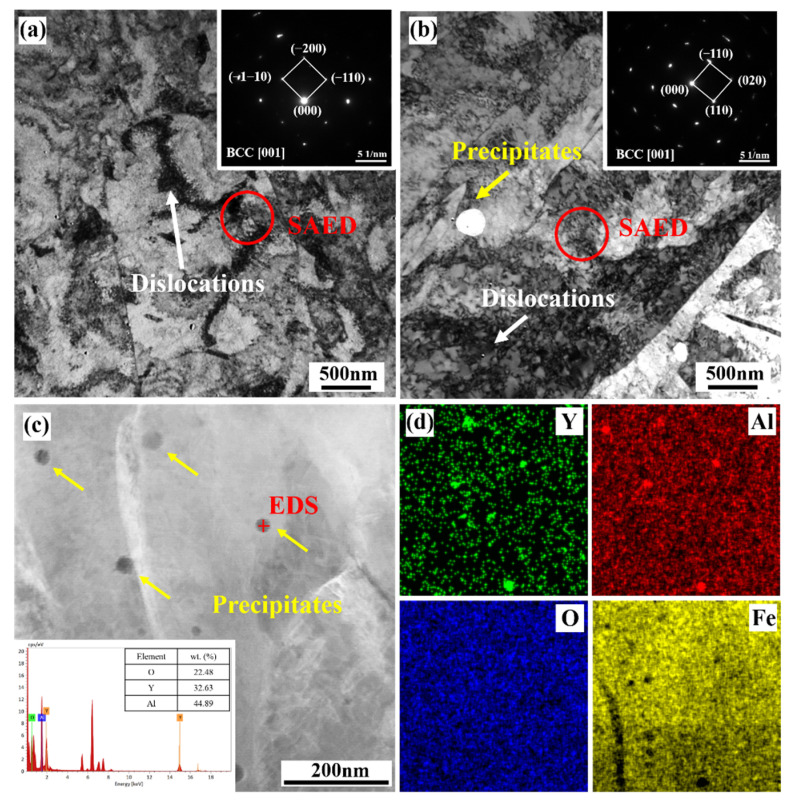
(**a**,**b**) TEM of heat-treated formed parts without and with yttrium, respectively; (**c**) TEM of the precipitates in yttrium-containing formed parts, with a point EDS analysis; (**d**) EDS mapping of the precipitates in yttrium-containing formed parts.

The precipitates in the stainless steel formed parts with and without Y were observed by transmission electron microscopy, as shown in Figure 6. It was found that in the field of view there was a high density of dislocations. The martensite strip with BCC structure was determined by selected area diffraction pattern (SAED) (Figure 6a,b). Spherical precipitates were only found in the parts with yttrium. A large number of previous studies [19,20] have shown that the precipitated phase in the traditional PH13-8Mo stainless steel is NiAl precipitate, which has a BCC structure and a good bonding performance with the stainless steel matrix. It is the main strengthening phase of PH13-8Mo stainless steel. However, the precipitation temperature of the NiAl phase is ~580 °C [25]. In this experiment, the temperature of 540 °C was applied for the aging treatment in order to avoid the generation of the NiAl phase. Therefore, no precipitation phase was expected in the formed parts without the rare earth yttrium. Thus, it can be reasonably deduced that these precipitates observed in the formed parts with yttrium were related to the addition of yttrium. Further observation of these precipitates (Figure 6c) showed that they were nearly spherical, with a size within 100 nm. Both point and mapping EDS analyses showed that these precipitates were rich in Y, Al and O.

### 3.3. Mechanical Performances

**Figure 7:**
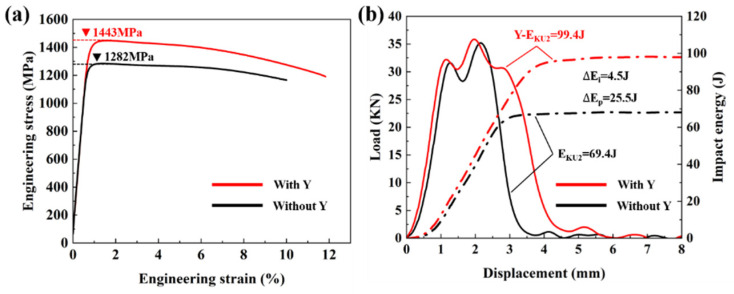
(**a**) Stress–strain curves of the formed parts with Y and without Y; (**b**) oscillographic impact curve of the shaped parts with different yttrium content.

Figure 7a,b respectively show the stress–strain curve and the oscillographic impact curve of heat-treated PH13-8Mo stainless steel formed parts with Y and without Y. Table 3 provides the corresponding data. From the tensile test data, the formed part with yttrium had a higher tensile strength, reaching 1443 MPa, i.e., 161 MPa higher than the that of the formed part without yttrium. At the same time, elongation was also improved. The elongation of the part with yttrium was 2% greater than that of the part without yttrium. According to the impact test data, the impact energy of the formed part without yttrium was 69.4 J. When yttrium was added, the impact energy of the formed part reached 99.4 J. In addition, in the oscillographic impact curve, the stage of impact energy fracture can be divided into crack formation success (E_i_) and crack propagation work (E_p_). Through the integral calculation of the curve, it was concluded that the changes in crack formation success and crack propagation work of the two formed parts were 4.5 J and 25.5 J, respectively. In order to eliminate the influence of the sample size, the impact energy value was normalized. The impact energy of the formed part without yttrium was 86.75 J/cm^2^, and that of the formed part with yttrium was 124.25 J/cm^2^. It can be seen that the effect of yttrium on the impact process of stainless steel was mainly evident in the crack propagation stage. Based on the tensile and impact test results, it can be concluded that adding the rare earth yttrium to PH13-8Mo stainless steel formed by SLM can improve the strength and toughness of the formed parts, especially the toughness. Generally, the mechanical properties of stainless steel will be balanced, that is, increasing strength will be balanced by decreasing toughness. However, in this work, a coordinated improvement of strength and toughness was achieved, effectively overcoming the problem of the strength–-toughness conflict present in previous works.

**Figure 8:**
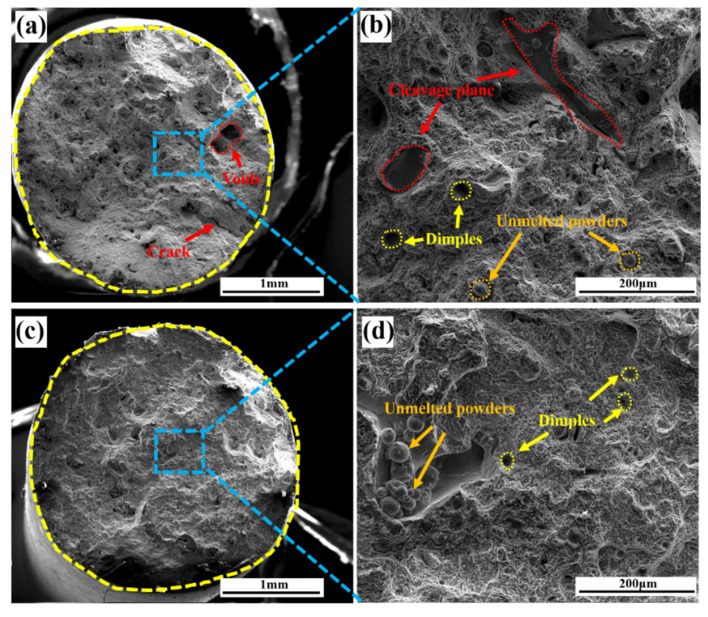
(**a**,**b**) Tensile fracture morphology of the formed parts without yttrium; (**c**,**d**) tensile fracture morphology of the formed parts containing yttrium.

Figure 8 shows the tensile fracture morphology of stainless steel formed parts with different yttrium content. It can be seen in Figure 8a,c that the two tensile parts presented a certain degree of contraction after fracture, showing the characteristics of ductile fracture. In Figure 8a, there are obvious voids and cracks on the fracture surface of the tensile part without yttrium. In the enlarged Figure 8b, a small amount of unmelted powder and small-size dimples are visible. However, some dissociation surfaces are also present, which indicates that some brittle fracture regions were formed in the fracture process. Figure 8c shows no obvious crack or void defect on the fracture surface of the tensile part containing yttrium, and no dissociation surface is found in the locally enlarged image in Figure 8d; mainly a small amount of unmelted powder and dimples are observed; the dimple size is also slightly larger than that of the tensile part without yttrium.

## 4. Discussion

Based on the phenomena observed in previous works and existing research results, we attribute the strengthening and toughening mechanism of yttrium on SLM-formed PH13-8Mo stainless steel parts to grain refinement, phase structure transformation and precipitated phase strengthening.

It can be seen in Figure 4 that the average grain size of the formed parts without and with Y was 2.02 ± 0.03 μm and 0.97 ± 0.02 μm, respectively. According to the Hall–Petch formula [26]

(1) $$\sigma_{gb}=\sigma_{0}+k_{H-P}d^{-1/2}$$

where the internal friction stress of body-centered cubic iron is $\sigma_{0}=50\;\mathrm{MPa}$ [27], the Hall–Petch coefficient of martensitic lath is $k_{H-P}=0.2{\;\mathrm{MPa}\;m}^{\frac{1}{2}}$ [20]. Therefore, it can be calculated that the increment of the strength of stainless steel with the addition of yttrium was $\Delta \sigma_{gb}=63.07\;\mathrm{MPa}$.

The effect of yttrium on the transformation of phase structure was mainly due to its influence on the content of residual austenite in PH13-8Mo stainless steel parts. It can be seen in Figure 5 that the addition of yttrium increased the residual austenite content in the formed parts from 17% to 20%. Martensite belongs to the BCC structure phase, and austenite belongs to the FCC structure phase in stainless steel. Martensite has high strength but poor toughness, belonging to a hard brittle phase [28,29]. Compared with martensite, austenite has lower strength, but it has excellent plastic toughness and is a ductile phase [30,31]. The rare earth yttrium could effectively improve the toughness of the formed parts of PH13-8Mo stainless steels by adjusting the phase fraction, that is, by increasing the austenite content and reducing the martensite content.

**Figure 9:**
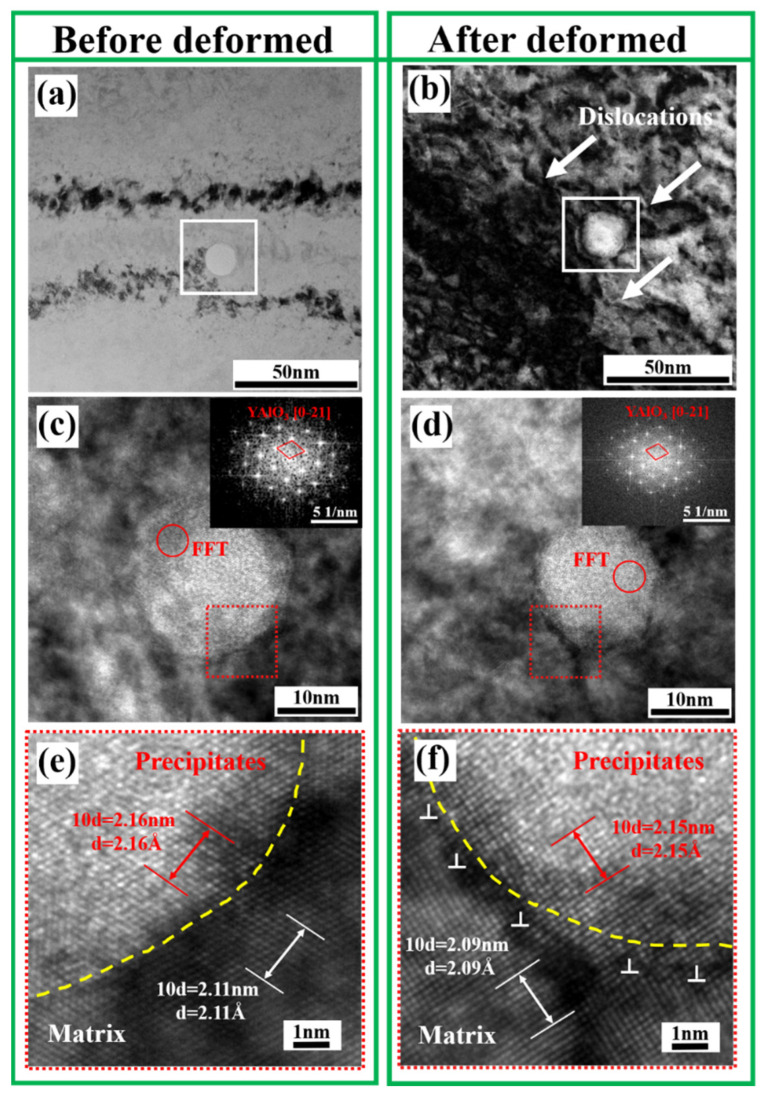
HRTEM results of nano-sized precipitates containing yttrium before and after deformation. (**a**,**c**,**e**) Precipitates before deformation; (**b**,**d**,**f**) precipitates after deformation.

Precipitate strengthening was due to the presence of nano-sized precipitates containing yttrium in the formed parts containing the rare earth yttrium after heat treatment, as shown in Figure 6. In order to explore the influence of the precipitates on the mechanical properties of the formed parts, a TEM comparison of yttrium-containing tensile parts before and after deformation was carried out, as shown in Figure 9. It was found that the precipitate before deformation was nearly spherical, with a size of about 15 nm. After Fourier transform calibration, we found that the precipitated phase was YAlO_3_. There were no obvious dislocations around the precipitated phase, and the rest of the matrix showed good adhesion. From the high-resolution TEM observation of the interface, it was seen that the mismatch between the precipitated phase and the matrix was ~2.3%, which conforms to the complete coherent relationship. Figure 9b,d,f shows the transmission diagram of the precipitates after deformation. It can be seen in Figure 9b that a large number of dislocations accumulated around the precipitates after deformation, which obviously hindered the movement of the dislocations. After deformation, the precipitated phase was still YAlO_3_, and the morphology remained nearly spherical without obvious changes. The size was about 15 nm. Through high-resolution TEM observation of the interface between the precipitates and the matrix, we found that the mismatch between the precipitates and the matrix was 2.9%, which still met the completely coherent relationship. However, an obvious dislocation distribution could be observed at the interface. The results showed that the precipitates containing the rare earth yttrium played a key role in strengthening the precipitates in PH13-8Mo stainless steel parts.

Generally, the strengthening effect of nano-sized precipitates on the matrix can be attributed to the Orowan bypass mechanism [32]. The size of YAlO_3_ precipitate is about 10 nm, which meets the size required by the Orowan bypass mechanism in stainless steel. The component of the strengthening mechanism can be calculated by Humphreys’ modified equation [33]:

(2) $$\sigma_{part}=\frac{0.84Gb}{\lambda -d},\;\lambda =d\sqrt{\frac{\pi }{6f}}$$

where *G* is the shear modulus (GPa), *b* is the Burgess vector, *d* is the diameter of the precipitate, *λ* is the distance between particles, and *f* is the volume fraction of the precipitate. According to the literature [34], the shear modulus of matrix is *G* = 94.1 GPa, *b* is taken as 0.248 nm, the diameter of the precipitation phase is 15 nm, and the volume fraction is 0.0026%. It was calculated that the strengthening component of yttrium-containing precipitates relative to the matrix was 100.8 MPa.

## 5. Conclusions

In this paper, two kinds of PH13-8Mo stainless steel powders without yttrium and with yttrium were prepared by the VIGA method and then were printed by SLM technology. Through the characterization of microstructure and mechanical properties of two kinds of shaped parts with different yttrium content, the following conclusions were reached:

(1) The addition of yttrium made the grains of PH13-8Mo stainless steel parts refined, and the average grain size from 2.02 ± 0.03 μm decreases to 0.97 ± 0.02 μm. PH13-8Mo stainless steel formed parts were mainly composed of martensite and retained austenite. After adding yttrium, the content of austenite in the phase composition increased, and yttrium-containing precipitates formed.
(2) The strength and toughness of the PH13-8Mo stainless steel parts were synergistically improved by adding yttrium. The tensile strength was increased from 1282 MPa to 1443 MPa, and the impact energy was increased from 86.75 J/cm^2^ to 124.25 J/cm^2^.
(3) The strengthening and toughening mechanism of yttrium on PH13-8Mo stainless steel parts was mainly attributed to grain refinement, phase structure transformation and precipitated phase strengthening. The nano-sized precipitates containing yttrium showed good adhesion with the matrix and played a role in preventing dislocation movement in the process of plastic deformation.

## Figures and Tables

**Table 1:** Chemical composition of PH13-8Mo stainless steel powders (wt.%).

	C	Si	Cr	Ni	Mo	Al	N	O	Y	Fe
Powder-0	0.039	0.019	12.59	8.28	2.24	0.99	0.0015	0.021	0	Bal
Powder-Y	0.039	0.017	12.56	8.23	2.23	0.94	0.0019	0.032	0.035	Bal

**Table 2:** SLM forming process parameters.

Laser Parameters	Value
Laser spot diameter	100 μm
Wavelength	1.06 μm
Hatch distance	0.08 mm
Layer thickness	0.03 mm
Laser power	200, 210, 220, 230, 240, 250, 260 W
Scanning speed	800, 850, 900, 950, 1000 mm/s

**Table 3:** Mechanical properties of the formed parts with Y and without Y.

	YS (MPa)	UTS (MPa)	TE (%)	IE (J/cm^2^)
Without-Y	1264	1282	10.2	86.75
With-Y	1399	1443	12.2	124.25

## Data Availability

The data presented in this study are available on request from the corresponding author.
